# Integrating Clinics, Laboratory, and Imaging for the Diagnosis of Common Variable Immunodeficiency-Related Granulomatous–Lymphocytic Interstitial Lung Disease

**DOI:** 10.3389/fimmu.2022.813491

**Published:** 2022-02-23

**Authors:** Marta Dafne Cabanero-Navalon, Victor Garcia-Bustos, Leonardo Fabio Forero-Naranjo, Eduardo José Baettig-Arriagada, María Núñez-Beltrán, Antonio José Cañada-Martínez, Maria José Forner Giner, Nelly Catalán-Cáceres, Manuela Martínez Francés, Pedro Moral Moral

**Affiliations:** ^1^ Primary Immune Deficiencies Unit, Department of Internal Medicine of the University and Polytechnic Hospital La Fe, Valencia, Spain; ^2^ Department of Pneumology, University and Polytechnic Hospital La Fe, Valencia, Spain; ^3^ Department of Radiology, University and Polytechnic Hospital La Fe, Valencia, Spain; ^4^ Department of Data Science, Biostatistics and Bioinformatics, Health Research Institute La Fe, Valencia, Spain; ^5^ Department of Internal Medicine, University Clinical Hospital, Valencia, Spain; ^6^ Department of Allergology, University and Polytechnic Hospital La Fe, Valencia, Spain

**Keywords:** GLILD, CVID, common variable immunodeficiency, interstitial lung disease, predictive model, diagnosis, scoring system, splenomegaly

## Abstract

**Background:**

Granulomatous–lymphocytic interstitial lung disease (GLILD) is a distinct clinic-radio-pathological interstitial lung disease (ILD) that develops in 9% to 30% of patients with common variable immunodeficiency (CVID). Often related to extrapulmonary dysimmune disorders, it is associated with long-term lung damage and poorer clinical outcomes. The aim of this study was to explore the potential use of the integration between clinical parameters, laboratory variables, and developed CT scan scoring systems to improve the diagnostic accuracy of non-invasive tools.

**Methods:**

A retrospective cross-sectional study of 50 CVID patients was conducted in a referral unit of primary immune deficiencies. Clinical variables including demographics and comorbidities; analytical parameters including immunoglobulin levels, lipid metabolism, and lymphocyte subpopulations; and radiological and lung function test parameters were collected. Baumann’s GLILD score system was externally validated by two observers in high-resolution CT (HRCT) scans. We developed an exploratory predictive model by elastic net and Bayesian regression, assessed its discriminative capacity, and internally validated it using bootstrap resampling.

**Results:**

Lymphadenopathies (adjusted OR 9.42), splenomegaly (adjusted OR 6.25), Baumann’s GLILD score (adjusted OR 1.56), and CD8+ cell count (adjusted OR 0.9) were included in the model. The larger range of values of the validated Baumann’s GLILD HRCT scoring system gives it greater predictability. Cohen’s κ statistic was 0.832 (95% CI 0.70–0.90), showing high concordance between both observers. The combined model showed a very good discrimination capacity with an internally validated area under the curve (AUC) of 0.969.

**Conclusion:**

Models integrating clinics, laboratory, and CT scan scoring methods may improve the accuracy of non-invasive diagnosis of GLILD and might even preclude aggressive diagnostic tools such as lung biopsy in selected patients.

## Introduction

Common variable immunodeficiency (CVID) constitutes a group of primary antibody deficiency disorders characterized by decreased IgG serum levels together with decreased IgA and/or IgM levels and reduced antibody response to immunization or infections (1). Nowadays, thanks to the introduction of immunoglobulin replacement therapy (IgRT), infectious diseases are not the main cause of morbidity and mortality in CVID patients. Non-infectious complications such as autoimmune or benign lymphoproliferative disorders (2, 3) have emerged as the comorbidities with a larger impact on prognosis and quality of life over infections involving up to 70% of patients (4). Nevertheless, their pathophysiology and the mechanisms by which they affect determined subsets of CVID patients are still poorly understood.

Granulomatous–lymphocytic interstitial lung disease (GLILD), a non-infectious lung complication, develops in 9% to 30% of patients with CVID (5, 6) and has been associated with long-term lung damage and poorer clinical outcomes in symptomatic patients (7, 8). After the Delphi consensus, GLILD has been defined as a distinct clinic-radio-pathological interstitial lung disease associated with lymphocytic infiltrate and/or granuloma in the lung and in whom other conditions have been considered and where possible excluded (9). Several radiological scoring systems for CT scans have recently been designed to evaluate the degree of lung injury in patients with CVID and to phenotype CT scans of GLILD patients (10, 11). However, the definite diagnosis requires both radiological and histopathological assessment of the lung, mainly through techniques such as transbronchial biopsy or video-assisted thoracoscopic (VATS) biopsy that entails important morbidity. Furthermore, it has been revealed as a major cause of death in CVID patients with non-infectious comorbidities owing to the lack of tools aiding its early clinical suspicion and diagnosis, as well as undefined therapeutic strategies (12). There is still no worldwide consensus on GLILD treatment and data on patients’ overall survival, and quantitative well-controlled evidence is lacking (12). Several immunosuppressants such as corticosteroids, cyclosporine, infliximab, azathioprine, and rituximab are being used, with variable efficacy (12, 13).

Some authors have demonstrated a significant association between GLILD and other dysimmune phenomena such as immune thrombocytopenic purpura (ITP), autoimmune hemolytic anemia (AIHA), and lymphatic hyperplasia of the spleen, liver, and lymphadenopathies (14–16). Similarly, ITP and AIHA have been related to the development of granulomas in any tissue (6, 17); and therefore, GLILD may not be exclusively considered as a localized form of lymphoproliferation, as it has been suggested in some works evaluating the features of the disease using ^18^F-FDG PET/CT (18, 19).

Further evidence is hence required on the independent predictors of GLILD in CVID (20). Less invasive alternative diagnostic approaches must be defined to reduce the morbimortality caused by lung biopsy and diagnostic delay, especially after diagnostic tools combining clinical, radiological, and analytical information are lacking.

The aim of this study was to explore the potential use of the integration between clinical and laboratory parameters associated with GLILD in patients with CVID, as well as develop CT scan scoring systems after external validation in order to improve the diagnostic predictability of non-invasive tools in a reference unit of primary immunodeficiencies.

## Material and Methods

### Study Design and Setting

A cross-sectional study was conducted *via* a retrospective review of electronic medical records of patients with a diagnosis of CVID in the Primary Immune Deficiencies Unit of the Department of Internal Medicine of the University and Polytechnic Hospital La Fe (UPHLF). UPHLF is a university hospital with 996 beds providing tertiary care in Valencia, Spain, and has an assigned population of 300,000. Patients with primary immunodeficiencies from the Valencian Community (population, 5,000,000) in the Spanish Mediterranean region are referred to the UPHLF Primary Immune Deficiencies Unit.

Patients with CVID diagnosis aged 18 years and above in follow-up by the Primary Immune Deficiencies Unit were considered eligible. These selected patients were screened to confirm CVID diagnosis according to the European Society for Immunodeficiencies (ESID) registry working definitions (21) for inclusion in the study. Cases were defined as patients with a chest high-resolution CT (HRCT) scan consistent with GLILD, a bronchoalveolar lavage excluding infectious pneumonia, and histological confirmation of GLILD after lung biopsy through both video-assisted thoracoscopic surgery (VATS) or transbronchial biopsy excluding malignancy (22). The final GLILD diagnosis was established after discussion in a multidisciplinary team involving a clinical immunologist, thoracic radiologist, pathologist, pneumologist, and internal medicine specialist in primary immunodeficiencies. Controls were defined as patients with CVID in the absence of interstitial lung disease. Patients with interstitial lung disease with no histological confirmation of GLILD or alternative diagnoses were excluded.

At the time of enrolment, both GLILD cases and controls were required to have at least one HRCT scan; IgG, IgA, IgM, low-density lipoprotein (LDL), high-density lipoprotein (HDL), and triglyceride levels at last follow-up prior to diagnosis or GLILD immunosuppressant therapy; one abdominal ultrasound; clinical history regarding cancer, enteropathy, autoimmune cytopenia, and lymphoproliferation; and B- and T-cell subpopulation count by flow cytometry, similar to previously described (16).

This study was approved by the Ethical Committee of Health Research Institute La Fe with registry code 2020-359-1 and was performed according to the Declaration of Helsinki.

### Data Collection

Several parameters were investigated for all patients:

- Demographic variables included sex, age, and date of diagnosis.
-Clinical parameters included type 1 diabetes mellitus, polyarthritis, dermatitis, hemolytic autoimmune anemia, ITP, Evans syndrome, generalized lymphadenopathies present at chest CT and/or CT-PET scans, splenomegaly (craniocaudal length >12 cm diagnosed after an abdominal ultrasound or CT scan), hepatopathy (elevation of liver enzymes, abnormal image findings in abdominal ultrasound or CT scan, or portal hypertension), frequent respiratory airway infections (≥3/year), enteropathy (chronic diarrhea or abnormal findings in a digestive tract biopsy), and presence of both solid and hematologic malignancy.
-Laboratory variables included IgG (mg/dl), IgM (mg/dl), IgA (mg/dl), LDL (md/dl), HDL (mg/dl), triglyceride levels (mg/dl), intake of lipid-lowering drugs recorded (statins, fibrates, NPC1L1 inhibitors, and PCSK9 inhibitors were considered), CD4 cell count (cell/µl), CD8 cell count (cell/µl), CD4/CD8 ratio, CD19 cell count (cell/µl), and natural killer (NK) cell count (cell/µl).
-Respiratory parameters included lung function tests (LFTs) such as forced expiratory volume in 1 s (FEV1), forced vital capacity (FVC), gas transfer [diffusing capacity of the lungs for carbon monoxide (DLCO) and corrected DLCO (cDLCO)], basal dyspnea measured by the Modified Medical Research Council (mMRC) scale, and chest CT findings of hilar lymphadenopathies, parenchymal lung disease, nodules, and bronchiectasis.
- Lung HRCT scan images and Baumann scoring method (11).
- Route and dosage of IgRT.

### High-Resolution CT Scan Collection and Analysis

The most recent available HRCT scan before treatment of GLILD with immunosuppressant agents was collected for each patient for image analysis. The exclusion criteria for assessment were as follows: presence of important motion artifacts, incomplete display of the lungs, and pneumothorax, as previously described (10).

HRCT scan images were analyzed by means of the software RadiAnt DICOM Viewer version 2021.1 (Medixant, Poznan, Poland), and the composite GLILD score of the Baumann Scoring method developed by the Chest CT Antibody Deficiency Group (11) was calculated for each patient. The scoring items included in the Baumann scoring method are represented in **Table 1**. In this scoring system, 13 different global lung image abnormalities and their extent are evaluated, creating a total of 22 scoring items per HRCT scan. The GLILD composite score is the sum of the following components: Number of lobes affected by ground glass + Ground glass due to fibrosis or inflammation + number of affected lobes with nodules smaller than 5 mm + number of affected lobes per nodules between 5 and 10 mm, + Number of affected lobes with frosted glass + frosted glass due to fibrosis or inflammation + number of affected lobes with nodules smaller than 5 mm + number of affected lobes with nodules between 5 and 10 mm + Number of Lobes Affected with Nodules Greater than 10 mm + Number of Lobes Affected with Reticulations + Predominance of Reticular Lines.

**Table 1:** Variables of Baumann’s scoring system.

Item	Definition	Score
**Number of lobes with:** Bronchial wall thickening Bronchiectasis Mucus large airways Mucus small airways Atelectasis Nodules <5 mm Nodules >5 to <10 mm Nodules >10 mm Lines Consolidation Linear scars and bands Ground-glass opacities Cysts Emphysema or bullae	Number of lobes	0–6
**Severity of:**		
Thickest bronchial wall thickening	0 = none 1 = BW < 0.5 × V 2 = 0.5 × V < BW < V 3 = BW > V	0–3
Largest bronchiectasis	0 = none 1 = B < 2 × V 2 = 2 × V < B < 3 × V 3 = B > 3 × V	0–3
**Pathologic mechanism of:**		
Cause ground-glass opacities	0 = fibrosis 1 = inflammation	0–1
Predominant type lines	0 = inflammation 1 = fibrosis 2 = mixed type	0–2
**Presence of:** Trapped air inspiratory scan Trapped air expiratory scan Lymphadenopathy hilar mediastinal	0 = no 1 = yes	0–1
**Size of:**		
Lymphadenopathy hilar mediastinal	Size (shortest axis) of largest lymph node	Millimeters

B, bronchial lumen; BW, bronchial wall; V, accompanying vessel.

Each HRCT lung scan was blindly analyzed by a specialized thoracic radiologist and a trained pneumologist.

### Statistical Analysis

The statistical analysis was conducted with the collaboration of the Department of Data Science, Biostatistics and Bioinformatics at Health Research Institute La Fe. The analyses were performed with the R statistical software version 4.0.5 (23).

Descriptive analyses were performed in order to summarize the patient characteristics. Quantitative data were expressed as mean and SD. Qualitative data were expressed as absolute count and percentage of cases. Univariate contrast analyses were only performed to check for epidemiological differences between both groups but not for predictor variables, as this type of analysis is subject to type I and II errors, as well as confusion bias. Significance was assessed by the χ^2^ or Fisher’s exact test for categorical variables and Student’s t-test for continuous variables with normal distributions, especially to ensure the lack of demographic differences between populations. Quantile–quantile plots were used to assess normality (**Figures S1, S2** in the **Supplementary Material**). A two-tailed p-value below 0.05 was considered statistically significant.

**Figure 1:**
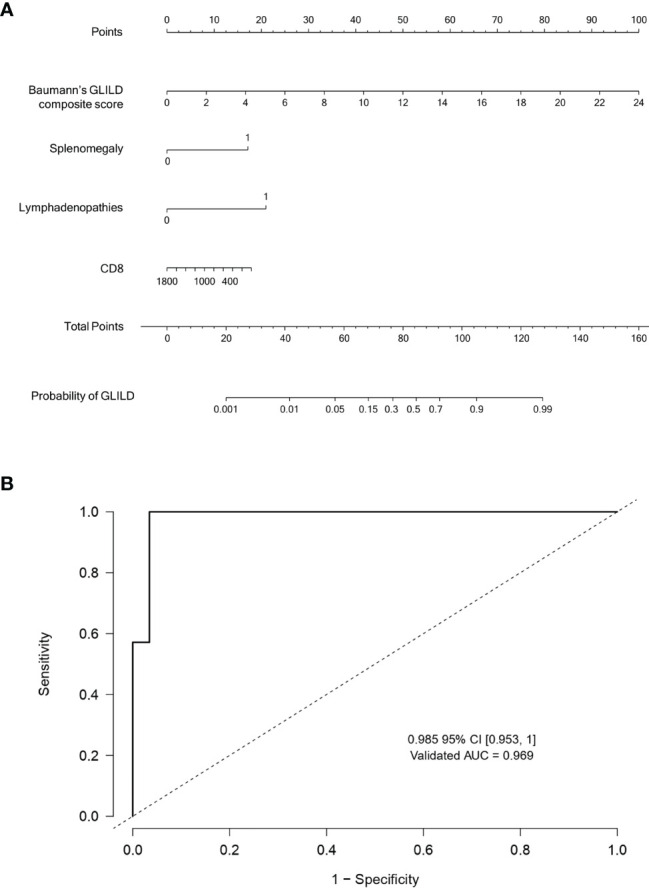
**(A)** Nomogram predicting the probability of GLILD in CVID patients. **(B)** Receiver operating characteristic curve of the Bayesian regression model including Baumann’s composite GLILD score, splenomegaly, CD8 cell count, and generalized lymphadenopathies for the prediction of GLILD. To obtain the nomogram predicted probability of GLILD, locate the patient values for each of the four variables at their own variable axis. Draw a vertical line to the upper “Point” axis to determine how many points are attributed for each variable value. Sum the points for all variables. Locate the sum of the total points in the “Total points” line. Draw a vertical line towards the “Probability of GLILD” line to determine the estimated probability of GLILD (an example of the use of this nomogram is provided in **Appendix S1**). GLILD, granulomatous–lymphocytic interstitial lung disease; CVID, common variable immunodeficiency.

Due to the low sample size inherent to the nature of the disease and high expected CIs, variables were selected according to previously published data and through the variable selection method *via* elastic net regularization using the *glmnet* package (24). Owing to the low number of observations with relatively high number of predictors, high collinearity, and dimensionality of the data, the elastic net technique was chosen. It outperforms both lasso and ridge methods and has been shown to serve as a variable selection technique, encouraging a grouping effect where strongly correlated predictors tend to be in or out of the model together (25). The amount of regularization was determined by the regularization parameter λ. Although assuming a lack of statistical significance due to high expected CIs, variables were included in the subsequent model. A Bayesian regression was performed with the final selected variables in order to jointly consider the relationship of the variables to each other in the prediction of the effect. The discriminative performance of the model was estimated by means of the area under the curve (AUC) of the receiver operating characteristic (ROC) curve (**Figure 1B**), and the best model including clinical, laboratory, and radiological parameters was selected. The internal validation of the model was assessed by a bootstrap technique using 500 generated bootstrap samples (26). Further information on the Bayesian model can be consulted in the **Supplementary Material**.

Finally, a nomogram was constructed to generate the graphic representation of the numerical probability of GLILD by integrating the final variables included in the model (**Figure 1A**).

To analyze the agreement in the measurements of Baumann’s GLILD composite score between both observers, Cohen’s kappa (κ) coefficient and CIs were calculated.

## Results

A total of 50 patients with CVID were enrolled in the study. Of the 50 CVID patients, 7 patients received a definite GLILD diagnosis and were considered as cases, and 2 patients with undefined interstitial disease pending biopsy and multidisciplinary assessment were excluded from the analysis. Only 2 controls underwent lung biopsy: one for severe bronchiectasis after lung transplant and the other one for etiological diagnosis of severe bilateral pneumonia without microbiological isolates, which finally showed *Pneumocystis jiroveci*. No significant differences were observed in age, sex, and diagnostic delay of both groups (Students t-test p-value = 0.128, Fisher’s test p-value = 1, Mann–Whitney test p-value = 0.593, respectively), although patients with GLILD diagnosis tended to be younger than control patients.

Thirty-seven out of 48 enrolled patients, with all GLILD cases included, underwent a genetic screening. Only one patient with GLILD presented a variant in PIK3R1 gene of uncertain significance. Among controls, 1 patient showed a pathologic mutation in NFKB2 gene, 1 patient had a pathological mutation in PIK3CD gene, 3 patients presented variants in NFKB1 gene (1 pathological and 2 possibly pathological), 1 patient had a non-pathological variant in RAG1 gene, and one patient presented a variant in TCF3 gene. Mutations in TCF3 gene have been related to immunodeficiencies and have been described in agammaglobulinemia with an autosomal dominant pattern of inheritance (27). The variant c.1555A>G found in our patient has not previously been described in CVID patients and has been previously reported in a healthy population with a very low frequency. After the application of several *in silico* predictive algorithms on the basis of evolutionary conservation, protein structure, protein function, and alignment and measurement of similarity between variant sequence and protein sequence homologs, it could only be interpreted as a variant of uncertain significance.

Further information on the genetic mutations or variants is described in **Table 2**.

**Table 2:** Description of the genetic mutations and variants.

Patient	Gene	Genetic mutation/variant	Protein change	Previously described in general population	Previously described in CVID patients	Interpretation
1 (GLILD)	PIK3R1 (heterozygosis)	c.5A>T	p.Tyr2Phe	Yes	No	Uncertain significance
2 (non-GLILD)	NFKB2 (heterozygosis)	c.2600_2619del	p.Ala867Glyfs*12	No	No	Pathological
3 (non-GLILD)	PIK3CD (homozygosis)	c.2608C>[	p.Arg870Ter	No	No	Pathological
4 (non-GLILD)	NFKB1 (heterozygosis)	c.94C>T	p.Gln32Ter	No	No	Pathological
5 (non-GLILD)	NFKB1 (heterozygosis)	c.983C>A	p.Ala328Asp	No	No	Possibly pathological significance
6 (non-GLILD)	RAG1 (heterozygosis)	c.1186C>T	p.Arg396Cys	Yes	No	Non-pathological^*^
7 (non-GLILD)	NFKB1 (heterozygosis)	c.920A>G	p.His307Arg	No	No	Possibly pathological significance
8 (non-GLILD)	TCF3 (heterozygosis)	c.1555A>G	p.Lys519Glu	Yes	No	Uncertain significance

CVID, common variable immunodeficiency; GLILD, granulomatous–lymphocytic interstitial lung disease.

*This mutation has been associated with severe combined immunodeficiency in homozygosis. The identification of this mutation in heterozygosis is not enough to explain the cause of the disease.

All patients were receiving IgRT. Six out of 7 GLILD patients and 31 out of 41 patients were under subcutaneous IgRT (ScIgRT). No differences were observed in the mean weekly Ig dose between both groups, being 8 and 11.69 g/week. None of the cases had smoking habits, and only one of the controls smoked.

Demographic parameters are summarized in **Table 3**.

**Table 3:** Patient characteristics.

Variable	CVID (n = 41)	GLILD (n = 7)
	Mean (SD)/n (%)	Mean (SD)/n (%)
Age	44.07 (15.74)	34 (17)
Male sex	17 (41.46)	3 (42.86)
Diagnostic delay of CVID (years)	11.08 (15.72)	8.43 (6.16)
Comorbidities		
Type I diabetes mellitus	2 (4.88)	1 (14.29)
Upper airway infections	26 (63.41)	3 (42.86)
Polyarthralgia	5 (12.2)	0 (0)
Skin disease	7 (17.07)	2 (28.57)
Autoimmune hemolytic anemia	2 (4.88)	4 (57.14)
Immune thrombocytopenic purpura	8 (19.51)	3 (42.86)
Evans syndrome	3 (7.32)	2 (28.57)
Lymphadenopathies	7 (17.07)	6 (85.71)
Splenomegaly	8 (19.51)	6 (85.71)
Liver affectation	4 (9.76)	1 (14.29)
Enteropathy	21 (51.22)	2 (28.57)
Malignant tumor	4 (9.76)	0 (0)
Lung parameters		
Lung function test		
Normal LFT	27 (79.41)	2 (33.33)
Obstructive LFT	6 (17.65)	1 (16.67)
Restrictive LFT	1 (2.94)	3 (50)
FEV1	95.18 (31.64)	87.5 (19.03)
FVC	103.69 (21.8)	90.17 (19.36)
cDLCO	79 (19.58)	70 (12.71)
Dyspnea degree (mMRC)		
0	33 (80.49)	6 (85.71)
1	4 (9.76)	1 (14.29)
3	1 (2.44)	0 (0)
4	3 (7.32)	0 (0)
Laboratory parameters		
Detected genetic mutation	8 (26.67)	1 (14.29)
IgG	965.33 (224.05)	1098.57 (192.17)
IgM	151.18 (425.79)	19.14 (25.22)
IgA	68.67 (204.88)	12.86 (20.81)
CD4	686.76 (482.42)	308.14 (132.89)
CD8	579.61 (412.06)	196.14 (88.48)
CD4/CD8	1.49 (0.91)	1.72 (1.01)
CD19	158.08 (139.45)	41 (54.75)
Natural killers	177.89 (109.2)	95 (52.68)
HDL cholesterol	50.56 (16.08)	43 (12.68)
LDL cholesterol	97.73 (34.95)	83.71 (25.32)
Triglycerides	93.68 (42.7)	124 (63.44)
Total cholesterol	167.22 (44.2)	151.57 (15.31)
Lipid-lowering drugs	0 (0)	5 (11)

cDLCO, corrected diffusing capacity of the lungs for carbon monoxide; FVC, forced vital capacity; FEV1, forced expiratory volume in 1 s; HDL, high-density lipoprotein; LDL, low-density lipoprotein; LFT, lung function test; CVID, common variable immunodeficiency; GLILD, granulomatous–lymphocytic interstitial lung disease; mMRC, Modified Medical Research Council.

### Baseline Clinical and Laboratory Parameters

Baseline comorbidities were analyzed in both groups at the time of inclusion. The absolute frequency of benign lymphoproliferative-related and autoimmune-related disorders was higher in the cases, as seen in **Table 3**. Namely, the percentage of patients presenting type 1 diabetes, hemolytic immune anemia, and immune thrombocytopenia—and hence, Evans syndrome—as well as splenomegaly and generalized lymphadenopathies was higher in patients suffering from GLILD than in control CVID patients with the Chapel “infection only” phenotype (7). However, the absolute frequency of recurrent airway infections, enteropathy, polyarthralgia, and malignancy tended to be increased in the control group (**Table 3**).

### Pulmonary Functional Assessment in Patients With Granulomatous–Lymphocytic Interstitial Lung Disease

Of the 7 patients with GLILD, two patients had undergone more than 1 LFT prior to diagnosis. Both of them presented significant moderate alterations: patient number 2 mainly progressed with the CO diffusing capacity being affected (decrease in 28% of the cDLCO). Patient number 5 showed both cDLCO impairment and a moderate restrictive ventilatory alteration through a reduction of 4.65% in the FVC from previously normal values in two sequential LFTs but with a known restrictive pattern due to a decreased total lung capacity (TLC) (68%) at the expense of decreased residual volume (RV) (38%).

Among the rest of the patients, 3 of them had undergone only 1 LFT before the time of diagnosis, and two of whom already presented clearly altered findings easily attributable to GLILD, namely, restrictive patterns and reduced cDLCO.

### Treatment and Follow-Up

Of the seven patients with GLILD, two of the patients presented functional and radiological progression in LFT prior to diagnosis. One received high-dose corticoid treatment with progressive descent to the minimum dose with poor response of both functional and image alterations, but without further significant progression, and the other was treated with mycophenolate following a 4-week rituximab regimen, with partial response and further stabilization. During the follow-up of the rest of the patients after diagnosis, two of them required treatment with azathioprine following rituximab in the same therapeutic scheme due to progression. A long-term response was achieved in one, but the other patient required two treatment cycles for relapse and further progression after a medium-term response. Three patients have not required treatment during follow-up to date.

### Inter-Observer Agreement of Lung Radiological Findings

**Figure 2:**
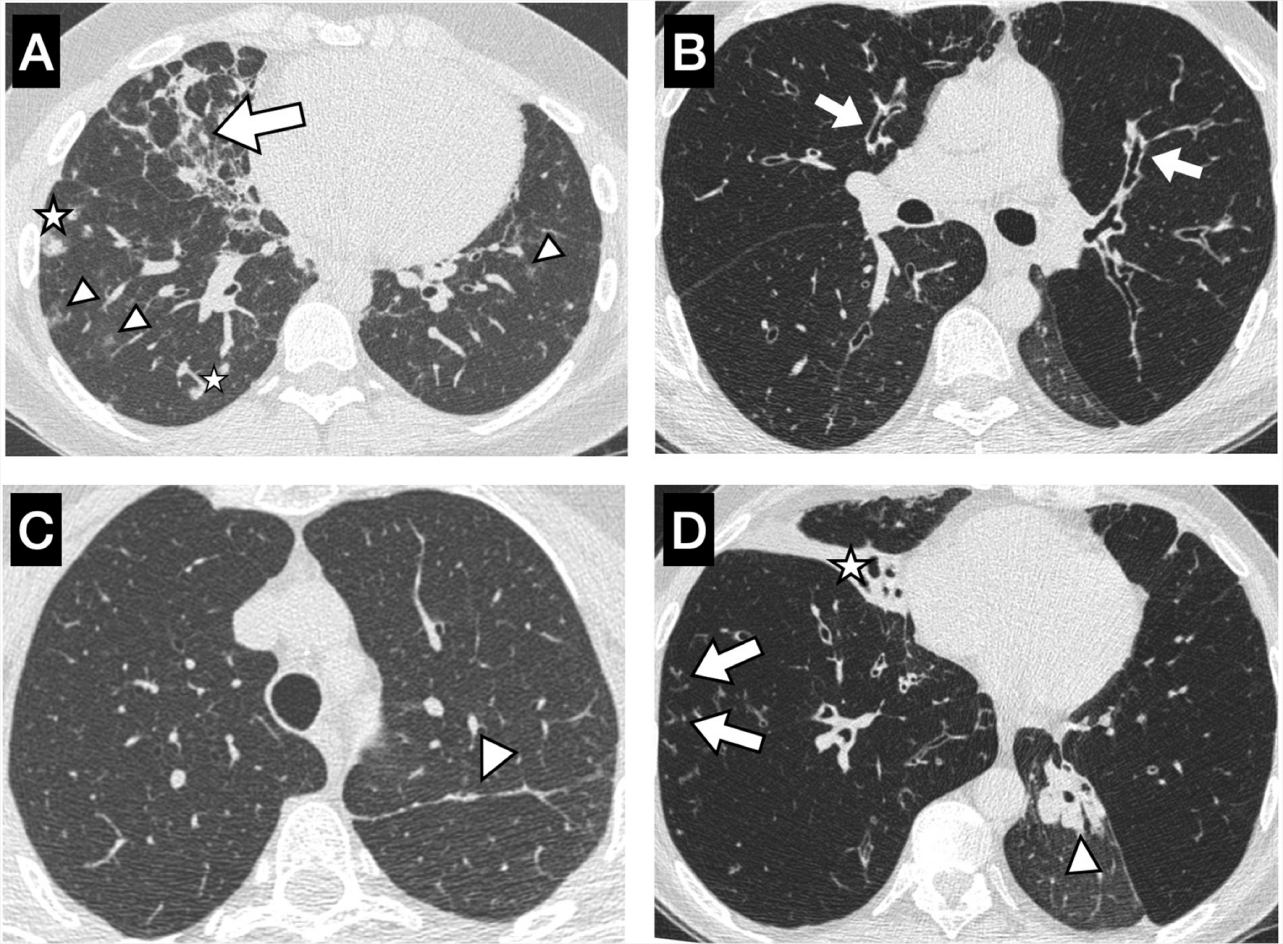
Chest CT from 4 different patients of our study with some of the evaluated parameters. **(A)** GLILD patient with multiple bilateral ground-glass nodules <5 mm (arrowheads), solid nodules (stars), and mixed (inflammatory and fibrotic) lines (arrow). **(B)** Non-GLILD patient with diffuse mild bronchial wall thickening and mild bronchiectasis (arrows) with air trapping predominantly in the upper lobes and partial atelectasis of left lower lobe. **(C)** Non-GLILD patient with linear parenchymal band in the left upper lobe (arrowhead). **(D)** Non-GLILD patient with mucus plugging in large airways of the left lower lobe with >50% atelectasis of the same lobe (arrows), complete atelectasis of the middle lobe (star), tree in bud pattern in the right lower lobe (arrows), and air trapping on the left upper and right lower lobes. GLILD, granulomatous–lymphocytic interstitial lung disease.

Bronchiectasis was present in 28.6% (2) of GLILD patients and in 44.7% (17) of controls, and their global presence in all patients who underwent an HRCT (n = 45) was 42.2%. Furthermore, 85.7% (n = 6) of cases showed hilar lymphadenopathies versus 24.4% (n = 10) in the control group. The number of lobes with nodules, reticulation, and ground-glass opacities after evaluation by both observers is represented in **Table 4**. Furthermore, the bronchiectasis and bronchial wall thickening scores, the airway disease composite score, and total Baumann’s score are represented in **Table 5**. Blinded Baumann’s GLILD composite scores were substantially higher for cases in both observers (**Table 4**). The mean GLILD score of both measures for GLILD patients was 17.07 (7), while it was 2.61 (3.72) for controls. Cohen’s κ statistic was 0.832 (95% CI 0.70–0.90), showing high concordance between the measures taken by the specialized thoracic radiologist and the trained pneumologist. Details on the radiological findings observed can be seen in **Figure 2**.

**Table 4:** Differences in the observers’ findings of variables included in Baumann’s composite score: nodules, reticulation, and ground-glass opacities, and composite GLILD score.

Variable	Observer 1 (pneumologist)		Observer 2 (thoracic radiologist)	
	Mean	SD	Mean	SD
Number of lobes with nodules >5 to <10 mm				
GLILD	3.66	2.58	3.80	3.03
Non-GLILD	0.00	0.00	0.18	0.55
Number of lobes with nodules >10 mm				
GLILD	0.50	0.84	1.25	1.89
Non-GLILD	0.03	0.18	0.03	0.19
Number of lobes with nodules <5 mm				
GLILD	4.66	2.33	5.40	1.34
Control	0.37	1.07	1.78	2.00
Number of lobes with reticulation				
GLILD	4.83	1.94	2.60	2.30
Non-GLILD	0.93	1.36	0.30	0.67
Number of lobes with GGO				
GLILD	4.33	2.16	1.60	2.07
Non-GLILD	0.50	1.11	0.48	1.40
GLILD score				
GLILD	19	9.27	15.14	4.74
Non-GLILD	2.32	3.04	2.9	4.41

GLILD, granulomatous–lymphocytic interstitial lung disease; GGO, ground-glass opacities.

**Table 5:** Differences in the observers’ findings in Baumann’s composite scores.

Variable	Observer 1 (pneumologist)		Observer 2 (thoracic radiologist)	
	Mean	SD	Mean	SD
Bronchiectasis				
GLILD	3.83	5.74	0.20	0.45
Non-GLILD	3.70	5.11	2.63	3.87
Bronchial wall thickening				
GLILD	9.66	5.64	1.40	2.60
Non-GLILD	7.66	6.24	2.63	4.43
Airway disease score				
GLILD	16.33	10.23	4.20	4.91
Control	13.96	11.84	7.11	9.78
GLILD score				
GLILD	19	9.27	15.14	4.74
Non-GLILD	2.32	3.04	2.9	4.41
Total score				
GLILD	45.16	14.21	99.40	45.92
Non-GLILD	17.96	15.04	95.93	39.04

GLILD, granulomatous–lymphocytic interstitial lung disease.

### Exploring Granulomatous–Lymphocytic Interstitial Lung Disease Associations Through Bayesian Regression

Several regression models were built through the elastic net variable selection technique including clinical, radiological, and laboratory parameters previously associated with GLILD in order to determine the set of interrelated variables with greater discrimination capacity. Mean Baumann’s composite GLILD score of both observers, splenomegaly, CD8 cell count, and generalized lymphadenopathies were selected by the elastic net model (regularization parameter λ of 0.1228). After inclusion in a Bayesian multivariable regression approach to explore their associations to GLILD, the presence of lymphadenopathies and splenomegaly was the most powerful predictor, with odds ratios (ORs) of 9.42 and 6.25, respectively. The OR of Baumann’s GLILD score was 1.56, and the CD8 cell count was found to be inversely related to the likelihood of GLILD (OR 0.9). However, as it can be seen in the constructed nomogram with the four variables (Baumann’s GLILD score, splenomegaly, CD8 cell count, and presence of lymphadenopathies) for the estimated probability of GLILD (**Figure 1**), the larger range of values of the validated Baumann’s GLILD scoring system gives it greater predictability.

This exploratory model was validated by calculating the AUC of the ROC curve (**Figure 1**), with a result of 0.985. The internal validation of the model in 500 generated bootstrap samples showed a very good discrimination capacity, with a validated AUC of 0.969.

## Discussion

The main findings of this study can be summarized as follows: i) the use of integrative predictive tools combining clinical, laboratory, and radiologic scoring parameters might be useful for the early diagnosis of GLILD in CVID patients. ii) The presence of splenomegaly, lymphadenopathies, low CD8 cell count, and high Baumann’s GLILD composite score predicts with almost perfect accuracy the presence of histologically confirmed GLILD even after internal validation. iii) Baumann’s GLILD score is strongly correlated to GLILD probability and is highly reproducible between trained observers.

Longitudinal cohort studies have not yet been developed to determine risk factors for GLILD, and there are no studies on the accuracy of predictive models combining clinical, analytical, and imaging variables for its diagnosis, mainly due to the nature of the disease. This is of remarkable importance, especially considering that GLILD has been defined as a distinct clinic-radio-pathological interstitial lung disease (9) and suggested to be the pulmonary component of a generalized dysimmune process in patients with CVID leading to a multisystemic lymphoproliferative disease (28). Furthermore, its definite histological diagnosis through lung biopsy carries an added burden of morbidity and mortality, with mortality rates for lung interstitial diseases reaching 1.7% in elected procedures (29). Here we present a first study integrating comorbidities and laboratory findings previously associated with the GLILD phenotype, including Baumann’s CT scoring system after external validation in our cohort, combining robust elastic net and Bayesian regression techniques. Despite its exploratory purpose and limited sample size from a reference unit of primary immunodeficiencies, this model demonstrates a very high discrimination capacity even after internal validation through the bootstrap technique that prompts replication in larger cohorts and multicenter studies.

However, this work presents obvious limitations. The small sample size due to the low prevalence of the disease and the single-center approach raises the probability of type II error, and the statistical potency is limited; therefore, this study was conceived exploratory in nature. As no prospective analyses have been performed to date, our work shares the limitation of a retrospective design with previous studies (14–16). Moreover, there may be a selection bias as a result of including patients from a tertiary care referral unit with potentially different characteristics from those of patients in the general population. The model also lacks an external validation with patients from other centers, subpopulations, and geographical origins. During the performance of this study and the collection time of the variables, none of the GLILD patients was actively treated with immunosuppressant agents. However, one of them had previously (6 years prior to GLILD diagnosis and variable collection) received a 4-week regimen of rituximab therapy due to benign generalized lymphadenopathies, and this fact must be considered, as it could influence some of the parameters in the model.

Beyond these constraints, several important concerns arise from the model. On the one hand, according to our data and as previously described, splenomegaly and lymphadenopathies are associated with GLILD (13–16). Both lymphadenopathies and splenomegaly are easily detectable by physical examination and CT scan, making these variables extremely useful in defining determined subsets of CVID patients who may develop GLILD or present with compatible image findings in clinical practice. These results also concur with the previously stated idea that GLILD is the lung expression of a systemic dysimmune lymphoproliferation disorder (28). This gains importance considering the difficulty of establishing a clinical suspicion based only on clinical symptoms, as patients are asymptomatic or present with completely unspecific symptoms, such as dyspnea on exertion or cough (12, 30). In fact, in our work, the degree of dyspnea was almost unchanged in both cases and controls (**Table 1**), with findings that contrast with the found alterations in LFTs (**Table 1**).

Almost 60% of our CVID patients with GLILD suffered from AIHA, while it was not observed in any of the control patients, as in the study by Chase and colleagues 2013 (13). A trend of lower absolute levels of IgA was also more frequently observed for cases. However, as in the work by Hartono and colleagues (14), it was not identified as an independent predictive factor itself, contrary to previous studies (13, 16). The prevalence of polyarthritis in both groups of patients was similar. In fact, none of our 7 cases suffered from arthralgia or arthritis, unlike previously suggested (15). Interestingly, the absolute frequency of patients with enteropathy seemed to be higher in the control group, which is striking, as it has been traditionally considered a non-infectious frequent complication possibly related to immune infiltration (19).

On the other hand, a lower CD8+ cell count appeared to be associated with the presence of histologically confirmed GLILD in the multivariable Bayesian model. Some studies have delved into peripheral blood lymphocyte subpopulations in characteristics in CVID patients with GLILD (16, 19, 31, 32). Our findings are in agreement with other reports in cellular defects in patients suffering from GLILD (16), autoimmune and granulomatous disorders (32), or interstitial lung disease (31), which also present an association between lower CD8+ subpopulations and the frequency of GLILD in that subgroup. Furthermore, and in concordance with our work, Bateman et al. (2012) (32) reported lower CD4+ and CD8+ lymphocyte counts in patients with CVID and GLILD compared to non-GLILD CVID patients. This was also documented by Kellner et al. (2019) (31) in their USIDNET Registry, with the most dramatic difference seen in the lower number of CD8+ cytotoxic T cells in patients with interstitial lung disease compared to those without lung disease. This finding could be related to a possible immune-mediated mechanism of lymphopenia, a frequent systemic comorbidity in patients with GLILD. Due to the limited sample size, the association of other cell compartments with GLILD cannot be firmly affirmed; however, a trend of lower CD19+ cell count was observed in the cases; and several authors previously stated that patients with splenomegaly, granulomas, enteropathy (33, 34), or interstitial lung disease (31, 33) had a lower B CD19+ cell compartment.

Recently, impaired HDL function has been linked to systemic inflammation in CVID patients with dysimmune complications (35). Therefore, we included for the first time its assessment in our GLILD cohort as well as other metabolic parameters such as LDL and TG. Despite that our results are limited, no absolute differences were observed between the lipid metabolism of cases and controls, and higher levels were not selected as protective factors in the model. Further studies with a larger sample size are needed to determine whether HDL function plays a role in the inflammatory pathways leading to the development of GLILD in CVID.

Interestingly, bronchiectasis was less frequently seen in our series than in other works in both cases and controls. While in the study by Meerburg and colleagues (10) bronchiectasis was present in 82% of all patients, the prevalence in our series was almost half, at 42%. Unlike previously reported (16), the absolute frequency of bronchiectasis was higher in the control group than in patients with GLILD. Additionally, the important differences in the higher prevalence of hilar lymphadenopathies in the cases advocate the idea that a common pathway of extrapulmonary lymphoproliferation may exist.

Several scoring systems have been developed for HRCT characterization in patients with CVID, such as the Baumann and Hartman methods (10, 11, 36). Despite that the Hartmann score has been reported to have slightly better inter-observer reproducibility for evaluating GLILD and airway disease, Baumann’s composite GLILD score was chosen in this study due to its easier application in clinical practice, good reproducibility for GLILD assessment, and lesser extensiveness. In our external validation of the Baumann score for GLILD prediction, our results improved those reported by Meerburg et al. (10), who showed an intraclass correlation coefficient of 0.74 and was similar to that observed for Hartmann’s GLILD composite score (0.84). We believe that this scoring system is a valuable outcome measure in research, but also its application in clinical practice and referral units might be useful for improving GLILD diagnostic accuracy.

To date, some works have been developed to identify potential predictors of GLILD that might aid in identifying subgroups of patients prone to developing GLILD or enhancing its clinical suspicion when facing compatible clinical symptoms and CT scan findings (14–16). However, GLILD is a clinical entity that has been recently redefined. Evidence is still scarce, and studies, despite some having large sample sizes, are not exempt from limitations. On the one hand, previous works include both histologically diagnosed and non-diagnosed patients as GLILD cases. As pathological confirmation in patients with clinical, analytical, and radiological suspicion is considered the gold standard for diagnosis, it could represent a selection bias, particularly including false positives, and could limit their result interpretation, extrapolation, and external validity. Therefore, in this study, only histologically confirmed GLILD cases after lung biopsy were included. In addition, like this work, all of them have a retrospective approach, but none has performed any internal or external validation, and despite carrying out univariate analyses with multiple comparisons, no p-value penalization is applied, resulting in an inflated type I error. Moreover, in the works by Mannina (15) and Cinetto (16), univariate analysis is used as a variable selection method for their inclusion in the regression model, which is a questionable approach since univariate analyses are less comprehensive and do not consider inter-variable relationships, making the final model prone to exclusion of false negatives, the inclusion of false positives, and important confusion biases.

In conclusion, our findings suggest the potential of models integrating clinics, laboratory, and CT scan scoring methods to improve the accuracy of non-invasive diagnosis of GLILD, which, after further research and validation, might even preclude aggressive diagnostic tools such as lung biopsy in selected patients. Additionally, these results highlight the need for ideally prospective and larger studies in order to unravel the pathophysiology of GLILD as well as develop diagnostic tools, allowing early diagnosis and leading to better long-term outcomes.

## Data Availability Statement

The raw data supporting the conclusions of this article will be made available by the authors, without undue reservation.

## Author Contributions

MC-N, VG-B, LF-N, and PMM conceived the idea, searched the bibliographic materials, and reviewed the existing literature. MC-N, VG-B, and PMM developed the figures and wrote the article. LF-N and EB-A analyzed the HRCT. AC-M and VG-B carried out the statistical analysis. MN-B, NC-C, MMF, and MFG reviewed the literature and contributed to the writing of the article. PMM supervised the work. MN-B and PMM are responsible for the care of the patients. All authors provided critical feedback and helped shape the final version of the manuscript.

## Funding

The article processing charge for publication has been funded by CSL Behring. The funding company has not intervened in the development of the project.

## Conflict of Interest

The authors declare that this study received funding from CSL Behring Ltd. The funder had the following involvement with the study: funding the article processing charge.

The authors declare that the research was conducted in the absence of any commercial or financial relationships that could be construed as a potential conflict of interest.

## Publisher’s Note

All claims expressed in this article are solely those of the authors and do not necessarily represent those of their affiliated organizations, or those of the publisher, the editors and the reviewers. Any product that may be evaluated in this article, or claim that may be made by its manufacturer, is not guaranteed or endorsed by the publisher.
